# Risk factors for household food insecurity in the Eastern Caribbean Health Outcomes Research Network cohort study

**DOI:** 10.3389/fpubh.2023.1269857

**Published:** 2023-11-23

**Authors:** Josefa L. Martinez-Brockman, Amber Hromi-Fiedler, Deron Galusha, Carol Oladele, Lisbette Acosta, O. Peter Adams, Rohan G. Maharaj, Cruz M. Nazario, Maxine Nunez, Marcella Nunez-Smith, Rafael Pérez-Escamilla, on behalf of the ECHORN Writing Group

**Affiliations:** ^1^Equity Research and Innovation Center, Yale School of Medicine, New Haven, CT, United States; ^2^Department of Internal Medicine, Yale School of Medicine, New Haven, CT, United States; ^3^Department of Social and Behavioral Sciences, Yale School of Public Health, New Haven, CT, United States; ^4^Department of Family Medicine, Faculty of Medical Sciences, University of the West Indies, Cave Hill, Cave Hill, Barbados; ^5^Department of Paraclinical Sciences, University of the West Indies, Saint Augustine, Trinidad and Tobago; ^6^Department of Biostatistics and Epidemiology, Graduate School of Public Health, University of Puerto Rico at Medical Sciences Campus, San Juan, Puerto Rico; ^7^School of Nursing, University of the Virgin Islands, St. Thomas, US Virgin Islands

**Keywords:** food insecurity, household food insecurity, Caribbean region, U.S. territories, ELCSA

## Abstract

**Background:**

Globally, 1.3 billion people were considered food insecure as of 2022. In the Caribbean region, the prevalence of moderate or severe food insecurity was 71.3% as of 2020, the highest of all subregions in Latin America. Experienced based measurement scales, like the Latin American and Caribbean Food Security Scale, are efficient measurement tools of food insecurity used globally. The Eastern Caribbean Health Outcomes Research Network (ECHORN) Cohort Study is a population-based longitudinal cohort study in the two Caribbean U.S. territories of Puerto Rico and the U.S. Virgin Islands, as well as in Barbados and Trinidad & Tobago. The purpose of this research was to examine the demographic, psychosocial, behavioral, and environmental risk factors associated with household food insecurity (HFI) among adults ≥40 years of age in the ECHORN cohort.

**Methods:**

A cross-sectional analysis of baseline ECHORN cohort study data was conducted. The primary outcome was household food insecurity (none, mild, moderate/severe). A total of 16 known and potential risk factors were examined for their association with HFI. The ANOVA and chi-square statistics were used in bivariate analysis. Ordinal logistic regression was used for the multivariable and sex stratified analyses.

**Results:**

More than one-quarter of the sample (27.3%) experienced HFI. In bivariate analyses, all risk factors examined except for sex, were significantly associated with HFI status. In the multivariable analysis, all variables except sex, education, marital status, smoking status, and residing in Puerto Rico were significant predictors of HFI in the adjusted model. In sex stratified analysis, depression, food availability, self-rated physical health, and island site were significantly associated with increased odds of worsening HFI for women, but not for men. Source of potable water was an important risk factor for both men and women.

**Discussion:**

The prevalence of HFI in the ECHORN cohort study is comparable to other studies conducted in the region. While women did not have an increased risk of HFI compared to men, a different set of risk factors affected their vulnerability to HFI. More research is needed to understand how water and food security are interrelated in the ECHORN cohort.

## 1 Introduction

The World Health Organization (WHO) defines food security as “a situation that exists when all people, at all times, have physical, social and economic access to sufficient, safe and nutritious food that meets their dietary needs and food preferences for an active and healthy life” (1). Globally, 1.3 billion people were considered food insecure as of 2022, with an increase of nearly 119 million people due to the pandemic in 2021 (2). Measuring food insecurity through household food insecurity (HFI) experience-based measurement scales continues to be the method of choice to assess food insecurity globally, compared to other methods like household expenditure surveys or dietary intake assessments (3). In adults worldwide, HFI has been associated with chronic diseases such as diabetes (4–6), hypertension (7) and overweight (8). In women and adults in low-income households, there is robust evidence of an association between HFI and malnutrition globally (8–13). Among children, HFI has been associated with childhood obesity (4, 14), stunting (15), malnutrition (15–18) as well as disability and/or injury (4).

In the Caribbean region, the prevalence of moderate or severe food insecurity was 71.3 percent in 2020, the highest of all subregions in Latin America when measured using the Food Insecurity Experience Scale (19). In this region, HFI has been associated with HIV/AIDS in Haitian adults (20, 21), HIV in adults in the Dominican Republic (22), and malnutrition in adults from both the Dominican Republic and Haiti (6, 22). HFI is associated with lower household income, physical disability, and having an underweight body mass index among adults in Trinidad & Tobago (23, 24). In Barbados, HFI is associated with disability and/or injury (4), and in Puerto Rico the Covid-19 pandemic worsened food insecurity in many households (24). In children in the Caribbean, HFI has been associated with child disability, family divorce or separation, and increased child healthcare needs in Caribbean households with children in the Eastern Caribbean Child Vulnerability Study (4). Among adolescents in a five-country study that included Trinidad & Tobago, HFI was associated with negative psychological and behavioral outcomes (25). In rural Haiti, HFI was associated with childhood malaria (26).

Household food insecurity must be considered in the context of water security (27–29). There is a consistent relationship between water and food insecurity. Indeed, in a study conducted in 27 sites in 21 low-and middle-income countries, the Household Water Insecurity Experiences (HWISE) Scale revealed an association between increasing rates of household water insecurity and decreasing availability and quality of food in the household (27, 28, 30). HFI is exacerbated by water insecurity through the direct limitation of food options that can be prepared due to a lack of potable water (31) and by directly limiting the budget for household food items due to the need to pay for treatment of potable water (28). Water insecurity is also associated with non-communicable diseases such as malaria, obesity, diabetes, and hypertension (32).

Existing cross-sectional and prospective epidemiologic studies that have examined risk factors for food insecurity are primarily focused on the United States or other high resource settings. In the U.S. these risk factors include having a lower level of education, never being married or being divorced/separated, being young, renting, or being African American or Hispanic (33, 34). Few epidemiologic studies exist that examine risk factors for food insecurity in the Caribbean region. The Eastern Caribbean Health Outcomes Research Network (ECHORN) Cohort Study is an ongoing population-based longitudinal cohort study designed to follow adults 40 years of age and older in the two Caribbean U.S. territories of Puerto Rico and the U.S. Virgin Islands, as well as in the nations of Barbados and Trinidad & Tobago. Its primary purpose is to measure the prevalence and incidence of diabetes, cancer, and heart disease as well as known and potential risk factors including food insecurity. The Caribbean region has the highest burden of non-communicable diseases, compared to Latin America, the U.S., and Canada. In fact, the U.S. Caribbean territories of Puerto Rico and the U.S. Virgin Islands are home to nearly 3.4 million Americans, yet we know very little regarding the risk factors for HFI and the relationship between HFI and non-communicable diseases on these islands. ECHORN is the first multi-country, intergenerational cohort study in the region designed to examine non-communicable disease outcomes and their known and potential risk factors. The purpose of this research was to examine the demographic, psychosocial, behavioral, and environmental risk factors associated with household food insecurity among adults ≥40 years of age in the ECHORN cohort.

## 2 Methods

The ECHORN study protocol was reviewed and approved by the Institutional Review Boards at Yale University, the University of Puerto Rico Medical Sciences Campus, the University of the Virgin Islands, the University of the West Indies – Cave Hill, and St. Augustine (Trinidad) campuses, and the Ministry of Health of Trinidad and Tobago. All participants provided their fully informed consent prior to initiating study procedures. The current analysis was approved by the Data Access and Scientific Review committee of the ECHORN Cohort Study.

### 2.1 Sample

Eligible participants at baseline were 40 years of age and older, English or Spanish speaking, able to provide informed consent, non-institutionalized at the time of data collection, had reliable contact/residential information, were semi-permanent or permanent residents of the island for 10 or more years, and had no plans to permanently relocate in the next 5 years.

The sampling methodology for the baseline ECHORN cohort (*n* = 2,961) has been described in detail elsewhere (35). Briefly, in Trinidad, Puerto Rico, and Barbados, stratified multistage probability sampling was used to empanel the baseline cohort between 2013 and 2018. In the US Virgin Islands simple random sampling was used across the islands of St. Thomas St. Croix and Saint John. Participants visited a community assessment center, centrally located on each island site, for their baseline assessment. After informed consent was obtained, participants were asked to complete a health survey, a clinical assessment, and provide a blood sample for immediate testing to identify markers of disease. The health survey consisted of questions pertaining to health status and chronic disease history, health behaviors, diet, household food insecurity, access to health care, migration history, social support, health networks, neighborhood factors, and demographic information. The cross-sectional sample used in this analysis included all participants with household food insecurity data at baseline and non-missing values for the examined risk factors (*n* = 1,939).

### 2.2 Primary outcome

The primary outcome was household food insecurity as measured by the Latin American and Caribbean Food Security Scale (or ELCSA by its Spanish acronym) (34). The 9-item ELCSA scale for adults (Table 1) is a household-level experiential food security scale and is scored by assigning 1-point to each affirmatively answered yes/no question. Next, responses are divided into the following categories: food secure (score of 0), mild food insecurity (score of 1–3), moderate (4–6), and severe food insecurity (score of 7–9). Respondents with moderate and severe food insecurity scores (4–9) were grouped into a single category.

**Table 1:** Latin American and Caribbean household food security scale items.

Item #	Question During the last 3 months, because of lack of money or other resources:
1	Were you worried about running out of food?
2	Did your home run out of food at any time?
3	Were you or any other adult in your home unable to eat the kinds of nutritious foods that make people healthy?
4	Did you or any other adult in your home usually have to eat the same foods almost every day?
5	Was there any day that you or any other adult in your home skipped a meal because of lack of food?
6	Did any adult in your home eat less food than what they needed because there wasn’t enough food?
7	Was there any day when you or any other adult in your home felt hungry but did not eat because there wasn’t enough food?
8	Was there any day when you or any other adult in your home did not eat for a whole day or just ate once during the day because there wasn’t enough food?
9	Did you do things that you would have preferred not to do, such as begging or sending children to work, to get food?

### 2.3 Independent variables

Sixteen risk factors were chosen and examined based on existing literature and potential risk factors specific to this population, based on experience working in the region. Demographic factors included age at baseline interview (continuous), sex, level of education, perceived economic status, marital status, island site (Puerto Rico, USVI, Trinidad, or Barbados), home ownership status (Yes/No), and whether the participant had moved in the past year (Yes/No). Sex was measured on the baseline survey using the following question, “What sex were you at birth?” Educational attainment was measured using the question, “What is the highest year of school that you completed?” Responses were categorized into less than high school (or secondary school), high school graduate, some college, and college and higher. Perceived economic status was measured using an adapted version of the World Gallup Poll^®^ question: “Please look at this figure, with steps numbered from 1 at the bottom to 10 at the top. Suppose the top of the ladder represents the richest people of this island and the bottom represents the poorest people of this island. Taking into consideration your current personal situation, what is the number of the step on which you would place yourself?” Responses ranged from 1 poor to 10 high and were categorized into bottom, middle, and top quantiles. Marital status was measured by asking “What is your current relationship status” and responses categorized into married, single, separated/divorced, or widowed.

Psychosocial factors included were emotional support, and depression. Emotional support was measured using the PROMIS Emotional Support short form (36). Responses were dichotomized (Yes/No) as to whether each participant had a low emotional support score, meaning less than 12. Depression (Yes/No) was measured by the Patient Health Questionnaire (PHQ-2) (37, 38).

Behavioral factors included current smoking and self-reported physical health scores. Current smoking status (Yes/No) was measured using two variables: “Have you EVER smoked any tobacco product, such as cigarettes, cigars, or tobacco pipe? Yes/No. Those that answered Yes were asked “Do you still smoke cigarettes, cigars, or tobacco pipe regularly? By regularly we mean at least 20 cigarettes or 1 cigar or half an ounce sachet of loose tobacco per month.” The PROMIS Global Physical Health score was used to assess participant reported physical health (39, 40). The score ranges from 4 to 20, with 4 being poor health and 20 excellent health. The score was created using 4 items (Table 2).

**Table 2:** PROMIS Global Physical Health score (40, 41).

Item	Response options
1. In general, how would you rate your physical health? (Choose one)	Excellent (1), very good (2), good (3), fair (4), poor (5)
2. To what extent are you able to carry out your everyday physical activities such as walking, climbing stairs, carrying groceries, or moving a chair? (Choose one)	Completely (1), mostly (2), moderately (3), a little (4), not at all (5)
3. In the past 7 days, how would you rate your pain on average? (Reverse scored and categorized as follows: (0 = 5; 1,2,3 = 4; 4,5,6 = 3; 7,8 = 2; 9 = 1)	0 no pain to 9 worst pain imaginable
4. In the past 7 days, how would you rate your fatigue on average? (Choose one) (Reverse scored from numbers shown in parenthesis to the right)	Very severe (1), severe (2), moderate (3), mild (4), none (5)

Environmental factors included fruit and vegetable availability and quality, mode of transportation to the grocery store, and water source as a proxy for water security. Fruit and vegetable availability and quality were measured as follows: “Thinking about food resources in your neighborhood, how often are a large selection of fresh fruits and vegetables, excluding provisions, available in my neighborhood?” and “Thinking about food resources in your neighborhood, how often are the fresh fruits and vegetables in your neighborhood of high quality?” Responses were dichotomized into never/rarely/sometimes *or* usually/always. Mode of transportation to the grocery store was measured with a single item: “What is the most typical way you travel to the store for your groceries?” and responses were dichotomized: drive own car/ride with friend/family *or* take the bus/taxi/bike/walk. Water insecurity was measured by a single item asking about source of potable water: “What is the main source of water supply for members of your household? This item was used as a proxy for water insecurity. Responses were dichotomized as water secure (water piped into dwelling) and water insecure (water not piped into dwelling).

### 2.4 Analysis

Rasch modeling was used to assess the ELCSA scale’s psychometric properties since this was the first time the scale was being used in the ECHORN Cohort. The Rasch model is a 1-parameter item response model, a modeling technique that is consistently applied in studies using the ELCSA and other food insecurity scales (26, 41–43). RASCH modeling was completed using the full data set (*n* = 2,961). The model was run in the following ways: (1) On the full sample using all 9 ELCSA items; (2) on the full sample using 8 of 9 ELCSA items (removing number 9—begging); (3) on the sample from each island (using all 9 items and 8 items as above); (4) removing individual participants identified as outliers—both on the full sample using all 9 items and for each island site using the 9-item scale. Unidimensionality of the scale by island was further assessed using Differential Item Functioning (DIF). DIF analysis was performed to compare scale performance for each island to the full sample. Measure, Infit values, and differences in item performance were assessed by island site. A detailed description of the RASCH results can be found in Supplementary material.

Next, univariate and bivariate analyses were conducted to determine the prevalence of household food insecurity, describe the overall sample by each risk factor, and to examine the association between household food insecurity and each risk factor. Study variables were summarized using means and standard deviations or frequency distributions for the total sample and by level of household food insecurity. The analysis of variance and the chi-square test were used to examine the association between potential risk factor variables and household food insecurity. Tetrachoric and polychoric correlation coefficients were also examined to determine whether collinearity existed between specific study variables: education, perceived economic status, water supply (which may be a marker for economic status in the Caribbean region), and home ownership. Finally, multivariable ordinal logistic regression was then used to determine the association between household food insecurity (mild to moderate/severe) and each risk factor holding all other variables constant. The analysis was first conducted for the full sample, then stratified by sex as women are more likely to experience HFI than men (44).

## 3 Results

### 3.1 Rasch modeling results

**Figure 1:**
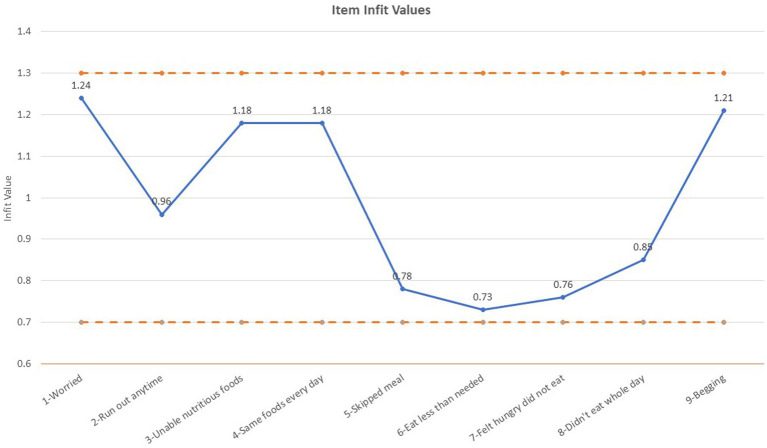
ELCSA item infit values.

Rasch modeling of the ELCSA scale in the ECHORN sample indicated that the full 9-item scale for adults used with the full cohort (rather than by island site) was the best fit. The Cronbach’s Alpha for the scale was 0.90. Figure 1 shows the ELCSA scale item infit values. Each item is shown along the X-axis and the item infit value on the Y-axis. Infit is a fit statistic that is less sensitive to outliers and more sensitive to observations near the respondent’s ability level (45). Acceptable infit values range from 0.7 to 1.3 (46). All 9-items of the ELCSA scale had acceptable infit values in the ECHORN sample. This means that the infit values demonstrate that the items measure the same construct and are independent of one another. Further information on the psychometric validity of the scale—as demonstrated through item prevalence, item severity, and differential item functioning by island site—is presented in Supplementary material.

### 3.2 Univariate and bivariate analysis

The final sample size consisted of 1,939 individuals with a baseline household food insecurity score and non-missing data for the examined risk factors. More than one-quarter of the sample (27.3%) experienced some level of household food insecurity (17.0% mild, 10.3% moderate or severe). Respondents were on average 57 years of age (S.D. 10.5) and nearly two-thirds were female. Nearly one-third had no high school education, 41% were married, nearly two-thirds owned their own home and 50% rated themselves in the middle economic quartile of their respective island. Nearly 15% of respondents did not have water piped directly into their homes (Table 3, total column). Correlation coefficients ranged from −0.04 to 0.23, indicating that collinearity was not present between education, perceived economic status, water supply, and home ownership.

**Table 3:** Risk factors for household food insecurity by HFI status (*n* = 1,939)^a^.

Characteristic	Total (*n* = 1,939)^b^	Food secure (*n* = 1,410)	Mild (*n* = 330)	Moderate/Severe (*n* = 199)	*p*-value^c^
Demographic factors					
Age (years)	57.2 (10.5)	58.7 (10.7)	53.9 (9.4)	52.4 (8.0)	<0.0001
Sex					0.5290
Male	673 (34.7)	499 (35.4)	111 (33.6)	63 (31.7)	
Female	1,266 (65.3)	911 (64.6)	219 (66.4)	136 (68.3)	
Education					<0.0001
No HS	626 (32.3)	430 (30.5)	120 (36.4)	76 (38.2)	
Completed HS	474 (24.5)	348 (24.7)	79 (23.9)	47 (23.6)	
Some college	436 (22.5)	301 (21.4)	80 (24.2)	55 (27.6)	
University degree	403 (20.8)	331 (23.5)	51 (15.5)	21 (10.6)	
Marital status					<0.0001
Married	793 (40.9)	618 (43.8)	123 (37.3)	52 (26.1)	
Single	787 (40.6)	531 (37.7)	145 (43.9)	111 (55.8)	
Separated/Div.	214 (11)	152 (10.8)	40 (12.1)	22 (11.1)	
Widowed	145 (7.5)	109 (7.7)	22 (6.7)	14 (7)	
Economic status					<0.0001
Bottom quartile	491 (25.3)	299 (21.2)	102 (30.9)	90 (45.2)	
Middle	979 (50.5)	723 (51.3)	171 (51.8)	85 (42.7)	
Top quartile	469 (24.2)	388 (27.5)	57 (17.3)	24 (12.1)	
Home ownership					<0.0001
No	661 (34.1)	380 (27)	156 (47.3)	125 (62.8)	
Yes	1,278 (65.9)	1,030 (73.1)	174 (52.7)	74 (37.2)	
Moved past year					<0.0001
No	1811 (93.4)	1,359 (96.4)	292 (88.5)	160 (80.4)	
Yes	128 (6.6)	51 (3.6)	38 (11.5)	39 (19.6)	
Island site					<0.0001
Barbados	553 (28.5)	437 (31)	70 (21.2)	46 (23.1)	
Puerto Rico	685 (35.3)	537 (38.1)	86 (26.1)	62 (31.2)	
Trinidad & Tobago	564 (29.1)	345 (24.5)	141 (42.7)	78 (39.2)	
US VI	137 (7.1)	91 (6.5)	33 (10)	13 (6.5)	
Psychosocial, behavioral, and environmental factors					
Low emotional support score (<12)					<0.0001
No	1706 (88)	1,288 (91.4)	274 (83)	144 (72.4)	
Yes	233 (12)	122 (8.7)	56 (17)	55 (27.6)	
Current smoking					<0.0001
No	1765 (91)	1,301 (92.3)	300 (90.9)	164 (82.4)	
Yes	174 (9)	109 (7.7)	30 (9.1)	35 (17.6)	
Depression					<0.0001
No	1,653 (85.3)	1,258 (89.2)	267 (80.9)	128 (64.3)	
Yes	286 (14.8)	152 (10.8)	63 (19.1)	71 (35.7)	
Physical health—mean, sd	14.4 (2.83)	14.7 (2.76)	14.0 (2.88)	13.4 (2.86)	<0.0001
Food availability					<0.0001
Never/rarely/sometimes	708 (36.5)	463 (32.8)	141 (42.7)	104 (52.3)	
Usually/always	1,231 (63.5)	947 (67.2)	189 (57.3)	95 (47.7)	
Food high quality					<0.0001
Never/rarely/sometimes	744 (38.4)	479 (34)	160 (48.5)	105 (52.8)	
Usually/always	1,195 (61.6)	931 (66)	170 (51.5)	94 (47.2)	
Mode of transport to get groceries					<0.0001
Drive own car/ride with friend or family	527 (27.2)	311 (22.1)	105 (31.8)	111 (55.8)	
Take the bus/taxi/bike/walk	1,412 (72.8)	1,099 (77.9)	225 (68.2)	88 (44.2)	
Water supply					
Not piped into dwelling	283 (14.6)	150 (10.6)	79 (23.9)	54 (27.1)	<0.0001
Piped into dwelling	1,656 (85.4)	1,260 (89.4)	251 (76.1)	145 (72.9)	

^a^Table values are mean ± SD for continuous variables and n (column %) for categorical variables; HS = High School.^b^Numbers may not sum to total due to missing data, and percentages may not sum to 100% due to rounding.^c^*P*-value is for *t*-test (continuous variables) and chi-square test (categorical variables).

In bivariate analyses, all risk factors examined, except for sex were significantly associated with household food security status (Table 3). Those who were food secure were 4.8–6.3 years older than those who had mild or moderate/severe food insecurity. Those who reported that they had not completed high school or college, were single, did not own a home, moved in the past year, or had a self-reported economic status in the bottom quantile were more likely to report food insecurity than their counterparts. Participants in Trinidad were more likely to report food insecurity compared to the other three island sites. The prevalence of water insecurity was 14.5% in this sample. A dose response relationship with water insecurity was found such that food insecurity worsened as the proportion of respondents who did not have potable water piped directly into their dwelling increased.

### 3.3 Multivariable analysis

The multivariable analysis modeled the odds of worsening household food insecurity (mild to moderate/severe; Table 4). Increasing age was protective against experiencing worsening HFI in this sample. All variables except sex, education, marital status, smoking status, and residing in Puerto Rico (compared to Barbados) were significant predictors of HFI in the adjusted model. Sex was not associated with HFI in either the unadjusted or the adjusted model. In the adjusted model, those who were depressed had 71% increased odds (OR: 1.71; 95% CI: 1.28–2.28) of worsening HFI compared to those who were not depressed. Those who did not have water piped directly into their dwelling had 59% increased odds of worsening HFI compared to those with water piped directly into their home (OR 1.59; 95% CI: 1.17–2.17).

**Table 4:** Unadjusted and adjusted associations between risk factors and household food insecurity (*N* = 1,939)*.

Variable	Unadjusted models		Adjusted model	
	OR	95% CI	OR	95% CI
Age (years)	0.95	0.94–0.96	0.95	0.94–0.96
Sex				
Male	1.00 (Reference)		1.00 (Reference)	
Female	1.12	0.91–1.38	1.03	0.81–1.32
Education				
No HS	2.12	1.57–2.88	0.83	0.57–1.22
Completed HS	1.69	1.22–2.33	0.87	0.60–1.26
Some college	2.11	1.52–2.91	1.36	0.95–1.94
University degree	1.00 (Reference)		1.00 (Reference)	
Marital status				
Married	1.00 (Reference)		1.00 (Reference)	
Single	1.77	1.41–2.21	1.22	0.94–1.57
Separated/Div.	1.45	1.04–2.03	1.42	0.97–2.07
Widowed	1.20	0.79–1.8	1.48	0.91–2.4
Economic status				
Bottom quartile	3.22	2.39–4.33	2.93	2.09–4.1
Middle	1.69	1.28–2.24	1.90	1.39–2.58
Top quartile	1.00 (Reference)		1.00 (Reference)	
Home ownership				
No	3.17	2.58–3.88	1.90	1.49–2.41
Yes	1.00 (Reference)		1.00 (Reference)	
Moved past year				
No	1.00 (Reference)		1.00 (Reference)	
Yes	4.53	3.23–6.36	2.34	1.59–3.43
Island site				
Barbados	1.00 (Reference)		1.00 (Reference)	
Puerto Rico	1.05	0.8–1.37	1.35	0.93–1.95
Trinidad and Tobago	2.29	1.76–2.98	1.92	1.39–2.65
US VI	1.79	1.19–2.68	1.99	1.21–3.29
Low emotional support				
No	1.00 (Reference)		1.00 (Reference)	
Yes	2.94	2.25–3.84	1.86	1.38–2.53
Current smoking				
No	1.00 (Reference)		1.00 (Reference)	
Yes	1.81	1.33–2.48	1.04	0.72–1.5
Depression				
No	1.00 (Reference)		1.00 (Reference)	
Yes	3.06	2.39–3.92	1.71	1.28–2.28
Physical health score	0.88	0.85–0.92	0.93	0.89–0.97
Food availability				
Never/rarely/sometimes	1.80	1.48–2.2	1.42	1.04–1.95
Usually/always	1.00 (Reference)		1.00 (Reference)	
Food high quality				
Never/rarely/sometimes	1.95	1.59–2.38	1.38	1.02–1.88
Usually/always	1.00 (Reference)		1.00 (Reference)	
Mode of transport to get groceries				
Drive own car/ride with friend or family	1.00 (Reference)		1.00 (Reference)	
Take the bus/taxi/bike/walk	2.66	2.16–3.28	2.40	1.84–3.13
Water supply				
Not piped into dwelling	2.73	2.13–3.51	1.59	1.17–2.17
Piped into dwelling	1.00 (Reference)		1.00 (Reference)	

*The Score Test for the proportional odds assumption for the adjusted model was not statistically significant (chi-square: 34.5582 (23 df); *p* = 0.06).

### 3.4 Sex stratified analysis

In the sex stratified analysis, age was a significant protective factor against worsening HFI; however, there was a greater protective effect for women compared to men (Table 5). For every 1-year increase in age, women were 6% less likely to experience worsening HFI, while men were 3% less likely. Self-reported economic status in the middle or bottom quantiles, not owning a home, having moved in the past year, low emotional support, lack of car or ride to get to the grocery store, and lack of water piped directly into the home were significantly associated with worsening HFI among both men and women. Education, marital status, current smoking status, and access to high quality foods were not associated with worsening HFI in men or women.

**Table 5:** Adjusted associations between risk factors and household food insecurity, stratified by sex (*n* = 1,939)*.

Variable	Males (*n* = 673)		Females (*n* = 1,266)	
	OR	95% CI	OR	95% CI
Age (years)	0.97	0.95–0.99	0.94	0.93–0.96
Education				
No HS	0.93	0.49–1.77	0.78	0.48–1.26
Completed HS	0.95	0.50–1.83	0.82	0.51–1.31
Some college	1.65	0.89–3.06	1.18	0.76–1.85
University degree	1.00 (Reference)		1.00 (Reference)	
Marital status				
Married	1.00 (Reference)		1.00 (Reference)	
Single	1.28	0.83–1.99	1.22	0.88–1.68
Separated/Div.	1.33	0.70–2.55	1.46	0.91–2.35
Widowed	0.91	0.17–4.82	1.66	0.98–2.82
Economic status				
Bottom quartile	3.18	1.72–5.86	2.99	1.99–4.52
Middle	2.11	1.19–3.75	1.84	1.27–2.66
Top quartile	1.00 (Reference)		1.00 (Reference)	
Home ownership				
No	1.85	1.22–2.8	1.92	1.43–2.59
Yes	1.00 (Reference)		1.00 (Reference)	
Moved past year				
No	1.00 (Reference)		1.00 (Reference)	
Yes	3.52	1.77–7.02	1.96	1.23–3.13
Island site				
Barbados	1.00 (Reference)		1.00 (Reference)	
Puerto Rico	1.37	0.74–2.55	1.37	0.86–2.18
Trinidad	1.66	0.94–2.93	2.12	1.43–3.14
USVI	1.13	0.48–2.68	2.99	1.59–5.61
Low emotional support				
No	1.00 (Reference)		1.00 (Reference)	
Yes	2.41	1.48–3.94	1.62	1.09–2.4
Current smoking				
No	1.00 (Reference)		1.00 (Reference)	
Yes	1.04	0.61–1.76	1.03	0.62–1.73
Depression				
No	1.00 (Reference)		1.00 (Reference)	
Yes	1.59	0.92–2.74	1.72	1.22–2.44
Physical health score	0.94	0.88–1.02	0.92	0.87–0.97
Food availability				
Never/rarely/sometimes	1.04	0.61–1.79	1.73	1.17–2.56
Usually/always	1.00 (Reference)		1.00 (Reference)	
Food high quality				
Never/rarely/sometimes	1.61	0.94–2.74	1.25	0.86–1.84
Usually/always	1.00 (Reference)		1.00 (Reference)	
Mode of transport to grocery store				
Drive own car/ride with friend or family	1.00 (Reference)		1.00 (Reference)	
Take the bus/taxi/bike/walk	2.02	1.26–3.23	2.66	1.92–3.67
Water supply				
Not piped into dwelling	1.87	1.08–3.21	1.49	1.01–2.19
Piped into dwelling	1.00 (Reference)		1.00 (Reference)	

*The Score Test for the proportional odds assumptions of each model were not statistically significant: Males—chi-square = 31.3249 (22 df); *p* = 0.09; Females—chi-square = 25.55 (22df); *p* = 0.27.

Depression, food availability, self-rated physical health, and island site were significantly associated with increased odds of worsening HFI for women, but not for men. Women who screened positive for depression had 72% increased odds of worsening HFI (OR: 1.72; CI: 1.22–2.44) compared to those without depressive symptoms. This is compared to 59% increased odds among men; however, this effect estimate was not statistically significant (OR: 1.59; CI: 0.92–2.74).

## 4 Discussion

This study aimed to examine demographic, psychosocial, behavioral, and environmental factors associated with HFI in a four-island Caribbean cohort. Identified demographic risk factors included younger age, lack of home ownership, and lack of stable housing. Psychosocial and behavioral risk factors included were depression, low emotional support, and poor self-rated physical health. Environmental risk factors included lack of food availability, lack of high-quality foods, and lack of water piped directly into the home. Our findings demonstrate that the prevalence of household food insecurity in the ECHORN Cohort is comparable to other studies that have been conducted in the region. A study of adults in Trinidad, showed a 25.0% prevalence of HFI and found that lower household income and physical disability were each independently associated with HFI (23). Another study conducted in households with children in three Eastern Caribbean countries (Barbados, St. Lucia, and St. Vincent and the Grenadines), that examined HFI as an exposure, showed a prevalence of HFI of 33.0% and found that food insecure households were more likely to include a chronically ill parent, among other factors (4). With respect to populations living with infectious diseases, the prevalence of HFI is even higher. Fifty-eight percent of people living with HIV in the Dominican Republic reported experiencing severe HFI (22). Finally, in a study of women with young children in Haiti, 98% of the sample had some level of food insecurity. This study found that severe food insecurity was a significant risk factor for clinical malaria (26).

The findings presented above differ from the existing literature in important ways. First, women are more likely to experience HFI than men, globally (47); however, sex was not associated with HFI in our bivariate or multivariable analyses. Given that female sex is a known risk factor for HFI in other regions of the world, a sex stratified analysis was conducted to understand how risk factors for HFI might differ by sex in this sample. In stratified analyses we found that women who screened positive for depression, had poorer self-rated physical health, and who did not think fresh fruits and vegetables were readily available had increased odds of worsening HFI. The existing literature demonstrates an association between HFI and depressive symptoms, anxiety, poor coping strategies, and risky behavior among women (48). The directionality of the association between depression and HFI is undetermined and while these findings do not directly fill that gap, they add to the body of literature demonstrating an association between mental health and HFI. Furthermore, women with poor self-rated physical health may have both physical and economic limitations that contribute to their food insecurity status. Future research should explore longitudinal associations between depression, self-rated physical health, food availability and HFI.

Furthermore, this study adds to the growing body of literature examining the association between water and food insecurity. Source of potable water-a proxy for water insecurity-was a significant predictor of HFI for both men and women, such that those without water piped directly into their home had an increased odds of experiencing HFI. We did not find evidence of multicollinearity between water source and other indicators of socioeconomic status such as education, perceived economic status, and home ownership, suggesting that source of potable water is an independent risk factor for HFI in this sample. To our knowledge, this is the first multi-country study in the Caribbean region to examine the association between water security and HFI. Recent scholarship on water and food security suggests collecting more and better data on water insecurity, including prevalence data (49). We will continue to explore the relationship between water and food security and corresponding health outcomes in subsequent waves of data collection for the ECHORN cohort.

### 4.1 Study strengths and limitations

We used a validated and well-tested measure of household food insecurity for this research and confirmed its robust psychometric properties in the ECHORN cohort. We also examined known and potential risk factors for HFI (based on our knowledge of the region), which allowed us to identify important risk factors specific to the populations under study. This research fills a gap in the literature with respect to identifying and understanding risk factors for household food insecurity in the Caribbean region, and strongly calls for applying the lessons learned in these settings to the design of similar policy relevant studies in other regions of the world. Importantly, we present evidence of a link between source of potable water, a proxy for water security, and household food insecurity in the ECHORN cohort. These findings have important implications for understanding how to improve the governance of food and water security systems and the coordination needed between them.

With respect to study limitations, the cross-sectional nature of this analysis only allows us to draw conclusions about the association between the studied risk factors and household food insecurity in the region, without comment on causality. In addition, these findings pertain to the ECHORN cohort study sites only and cannot be extrapolated to other nations or territories in region.

## 5 Conclusion

This cross-sectional, multi-country study was designed to identify risk factors for household food insecurity in the Eastern Caribbean. The findings fill a gap in the literature with respect to understanding risk factors for HFI and have important implications for future research and policy in this area. Future research should examine these risk factors longitudinally, with a focus on understanding the transition from a food secure to a food insecure state over time in the ECHORN cohort. Additional work will examine whether household food insecurity is associated with specific cardiometabolic conditions in the cohort and what role water security also plays in these relationships.

## Data availability statement

The datasets generated and analyzed for this study are available upon request to the ECHORN Data Access and Scientific Review committee (https://www.echorn.org/request-echorn-data).

## Ethics statement

This study was approved by the Institutional Review Boards at Yale University, the University of Puerto Rico Medical Sciences Campus, the University of the Virgin Islands, the University of the West Indies – Cave Hill, and St. Augustine (Trinidad) campuses, and the Ministry of Health of Trinidad and Tobago. This study were conducted in accordance with the local legislation and institutional requirements. The participants provided their written informed consent to participate in this study.

## Author contributions

JM-B: Conceptualization, Formal analysis, Methodology, Project administration, Supervision, Visualization, Writing – original draft, Writing – review & editing. AH-F: Formal analysis, Methodology, Software, Supervision, Writing – review & editing. DG: Data curation, Formal analysis, Methodology, Writing – review & editing. CO: Writing – review & editing. LA: Writing – original draft, Writing – review & editing. OA: Data curation, Funding acquisition, Resources, Writing – review & editing. RM: Data curation, Funding acquisition, Resources, Writing – review & editing. CN: Data curation, Funding acquisition, Resources, Writing – review & editing. MN: Data curation, Funding acquisition, Resources, Writing – review & editing. MN-S: Data curation, Funding acquisition, Resources, Writing – review & editing. RP-E: Conceptualization, Methodology, Supervision, Writing – review & editing.
