# Supplementary material for: Risk factors for household food insecurity in the Eastern Caribbean Health Outcomes Research Network cohort study
Source: Front Public Health. 2023 Nov 23;11:1269857. doi: 10.3389/fpubh.2023.1269857 (PMC10702572; doi:10.3389/fpubh.2023.1269857)
Supplement: Supplementary file 1 [file Data_Sheet_1.docx]

**Supplementary Material**

**Table 1. Proportion of Households that Responded Affirmatively to Each Question, ordered by level of severity (least to most), and Proportion of Households with each Raw Score (N=2,479) ^1^**

| **Households that Responded Affirmatively to Each Question** | | **Households with each Raw Score** | |
| --- | --- | --- | --- |
| **Adult/Household Item** | **n (%)^2^** | **Raw score^3^** | **n (%)^4^** |
|  | - | 0 | 1780 (60.1) |
| 1 – worried | 414 (14.0) | 1 | 235 (7.9) |
| 3 – unable to eat nutritious foods | 406 (13.7) | 2 | 126 (4.3) |
| 4 – same foods | 353 (11.9) | 3 | 79 (2.7) |
| 2 – run out of food | 269 (9.1) | 4 | 61 (2.1) |
| 6 – eat less food than needed | 253 (8.5) | 5 | 36 (1.2) |
| 5 – skipped a meal | 229 (7.7) | 6 | 59 (2.0) |
| 8 – didn’t eat entire day | 171 (5.8) | 7 | 41 (1.4) |
| 7 – felt hungry, did not eat | 170 (5.7) | 8 | 41 (1.4) |
| 9 - begging | 41 (1.4) | 9 | 21 (0.7) |

^1^The full baseline sample includes 2,961 participants; n=482 are missing all 9 baseline ELCSA scores because the scale was added to the baseline survey after enrollment began; ^2^Total does not sum to 2,479 due to missing data on individual items. ^3^Raw score is the cumulative number of items affirmed by each household; ^4^ 28.2% (699/2,479) of households had some level of food insecurity [mild (n=440), moderate (n=156), or severe (n=103); ]; 0 = secure; 1 – 3 = mild; 4-6 = moderate; 7-9 = severe

**Table 2. Tetrachoric Correlation Coefficients (Cronbach’s alpha 0.90)**

| Item # | 1 | 2 | 3 | 4 | 5 | 6 | 7 | 8 | 9 |
| --- | --- | --- | --- | --- | --- | --- | --- | --- | --- |
| 1 | 1.000 | 0.851 | 0.763 | 0.757 | 0.779 | 0.819 | 0.781 | 0.738 | 0.725 |
| 2 |  | 1.000 | 0.795 | 0.783 | 0.842 | 0.846 | 0.855 | 0.852 | 0.762 |
| 3 |  |  | 1.000 | 0.807 | 0.786 | 0.827 | 0.776 | 0.776 | 0.662 |
| 4 |  |  |  | 1.000 | 0.838 | 0.824 | 0.771 | 0.731 | 0.712 |
| 5 |  |  |  |  | 1.000 | 0.941 | 0.921 | 0.900 | 0.735 |
| 6 |  |  |  |  |  | 1.000 | 0.939 | 0.912 | 0.783 |
| 7 |  |  |  |  |  |  | 1.000 | 0.940 | 0.753 |
| 8 |  |  |  |  |  |  |  | 1.000 | 0.816 |
| 9 |  |  |  |  |  |  |  |  | 1.000 |

Item Prevalence values are shown in Figure 1 above. These value reflect the first n (%) column shown in Table 1 above, without being ordered by level of severity as in Table 1.

**Item Severity**

Item severity reflects the concepts of person ability and item difficulty such that a person of lesser ability is not expected to answer items of higher difficulty (1). The higher the item severity score, the more severe the item is and the less it is expected to be affirmed, especially by those of lesser ability.

As shown in Figure 2, item severity ranges from 0.19 to 6.05. Item severities for items 1-8 range between 0.19 and 2.74. The last question (#9) asks about whether the head of household had to beg for food or send children to work to get food, indicating a more severe level of household food insecurity. As expected, fewer participants respond affirmatively to this question (n = 41; Table 1 and Figure 1), indicating greater severity of the item.

**Cross-Island Comparison**

RASCH modeling was conducted on the full sample using the 9-item scale and for each island site using the 9-item scale. For each island site we also conducted RASCH analysis on the 9-item scale with outliers removed and on the 8-item scale, after removing question 9 (beg for food). Table 3 displays the item order, item severity, and infit for the full sample using the 9-item scale, and for each island site using the 9-item scale.

**Table 3. Item Order, Severity, and Infit by Island Site and Full Sample Using the 9-Item Scale***

| **Full Sample** | | | **Barbados** | | | **Trinidad** | | | **Puerto Rico** | | | **USVI** | | |
| --- | --- | --- | --- | --- | --- | --- | --- | --- | --- | --- | --- | --- | --- | --- |
| Ord. | Sev. | Infit | Ord. | Sev. | Infit | Ord. | Sev. | Infit | Ord. | Sev. | Infit | Ord. | Sev. | Infit |
| 9 | 4.05 | 1.21 | 9 | 3.94 | 1.15 | 9 | 4.57 | 1.27 | 9 | 3.82 | 1.1 | 9 | 3.76 | 1.28 |
| 7 | 0.75 | 0.76 | 7 | 0.7 | 0.73 | 7 | 0.57 | **0.67** | 8 | 1.73 | 0.74 | 8 | 0.85 | 0.95 |
| 8 | 0.74 | 0.85 | 8 | 0.42 | 0.88 | 8 | 0.41 | 0.78 | 7 | 1.22 | 0.89 | 7 | 0.6 | 0.86 |
| 5 | 0.02 | 0.78 | 5 | 0.33 | 0.78 | 5 | -0.29 | 0.78 | 5 | 0.31 | 0.7 | 2 | -0.08 | 0.76 |
| 6 | -0.35 | 0.73 | 6 | -0.3 | 0.77 | 2 | -0.38 | 1.02 | 6 | -0.21 | 0.74 | 5 | -0.16 | 0.88 |
| 2 | -0.42 | 0.96 | 2 | -0.54 | 1.01 | 6 | -0.52 | 0.74 | 2 | -0.59 | 1.01 | 6 | -0.24 | **0.62** |
| 4 | -1.24 | 1.18 | 4 | -1.08 | 1.24 | 4 | -0.7 | **1.33** | 3 | -1.09 | 1.21 | 1 | -1.45 | **1.34** |
| 3 | -1.73 | 1.18 | 3 | -1.59 | 1.19 | 1 | -1.33 | 1.23 | 4 | -2.17 | 0.88 | 3 | -1.53 | 1.16 |
| 1 | -1.81 | 1.24 | 1 | -1.89 | 1.15 | 3 | -2.33 | 1.15 | 1 | -3.02 | **1.34** | 4 | -1.75 | 1.14 |

*Ord. = Item order, Sev. = Item severity; values in **bold** are outside the acceptable infit range of 0.7 – 1.3

When the item infit values were examined within each country, we saw for each country across the 9 items, all fit well within the range, with the exception of the following (in bold in table above):

Trinidad: items 4 (eat the same food every day) and 7 (felt hungry but did not eat);

Puerto Rico: item 1 (worried about running out of food);

USVI: items 6 (eat less food than what needed) and 1 (worried about running out of food).

Based on the collected information we concluded that the 9-item scale analyzed using the full sample (rather than by island site) is appropriate here.

**Differential Item Functioning**

The differential item functioning measure is used to assess the difficulty/severity of each item for each group – in this case island site. The DIF score is estimated while holding constant all the other item difficulty/severity and person ability measures. The differential item functioning score is the difference between the DIF measure for a given class (i.e., island site) and the baseline difficulty measure, measured in logits (1). Our Differential Item Functioning analysis indicated that the use of the 9-item scale across the full cohort was appropriate (Figure 3).

**References**

1. Bond TG, and Fox, C.M. Applying the Rasch Model: Fundamental Measurement in the Human Sciences. Third Edition ed. New York, NY: Routledge; 2015.

**Figure 1. Item Prevalence**

**
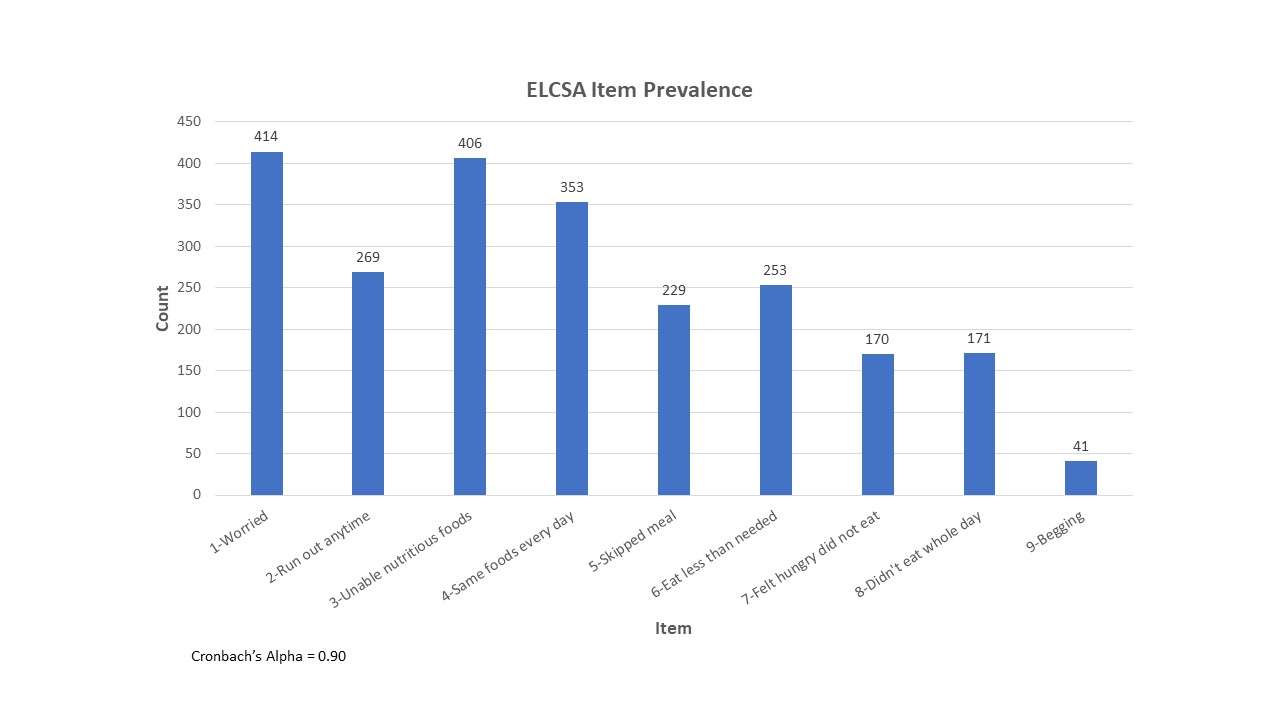
**

**Figure 2. Item Severity**

**
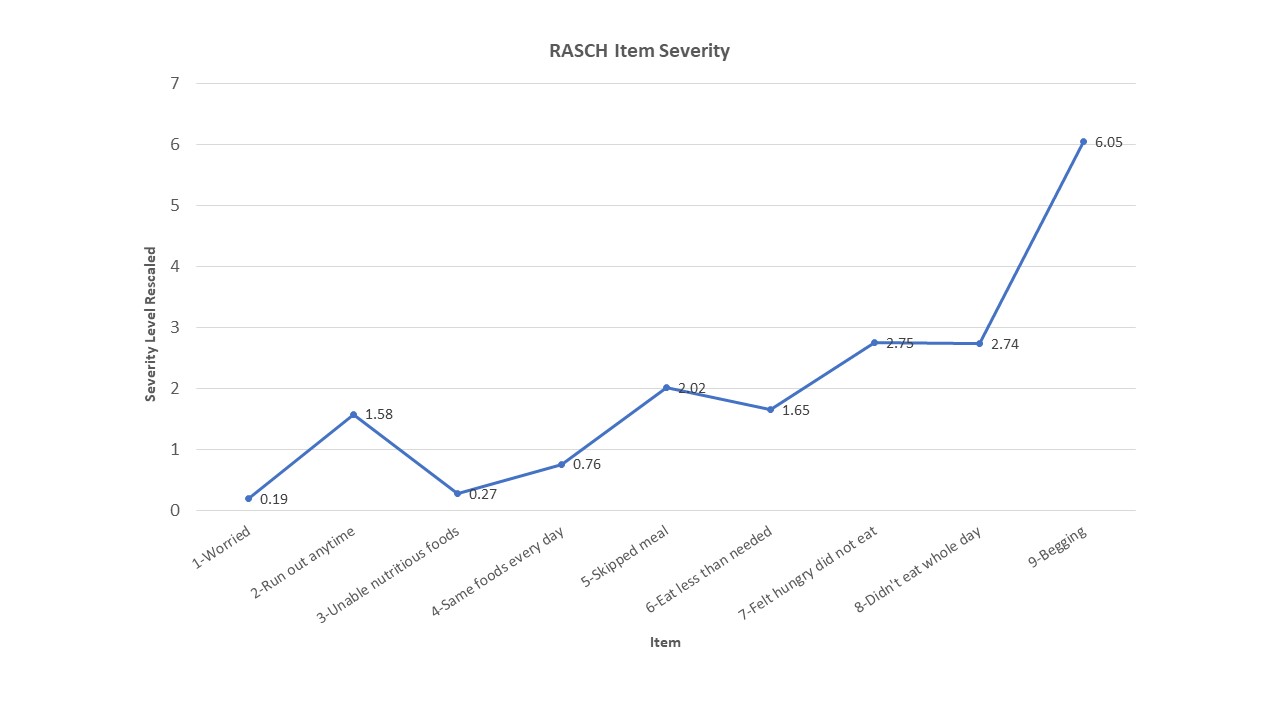
**

**Figure 3. Differential Item Functioning**

**
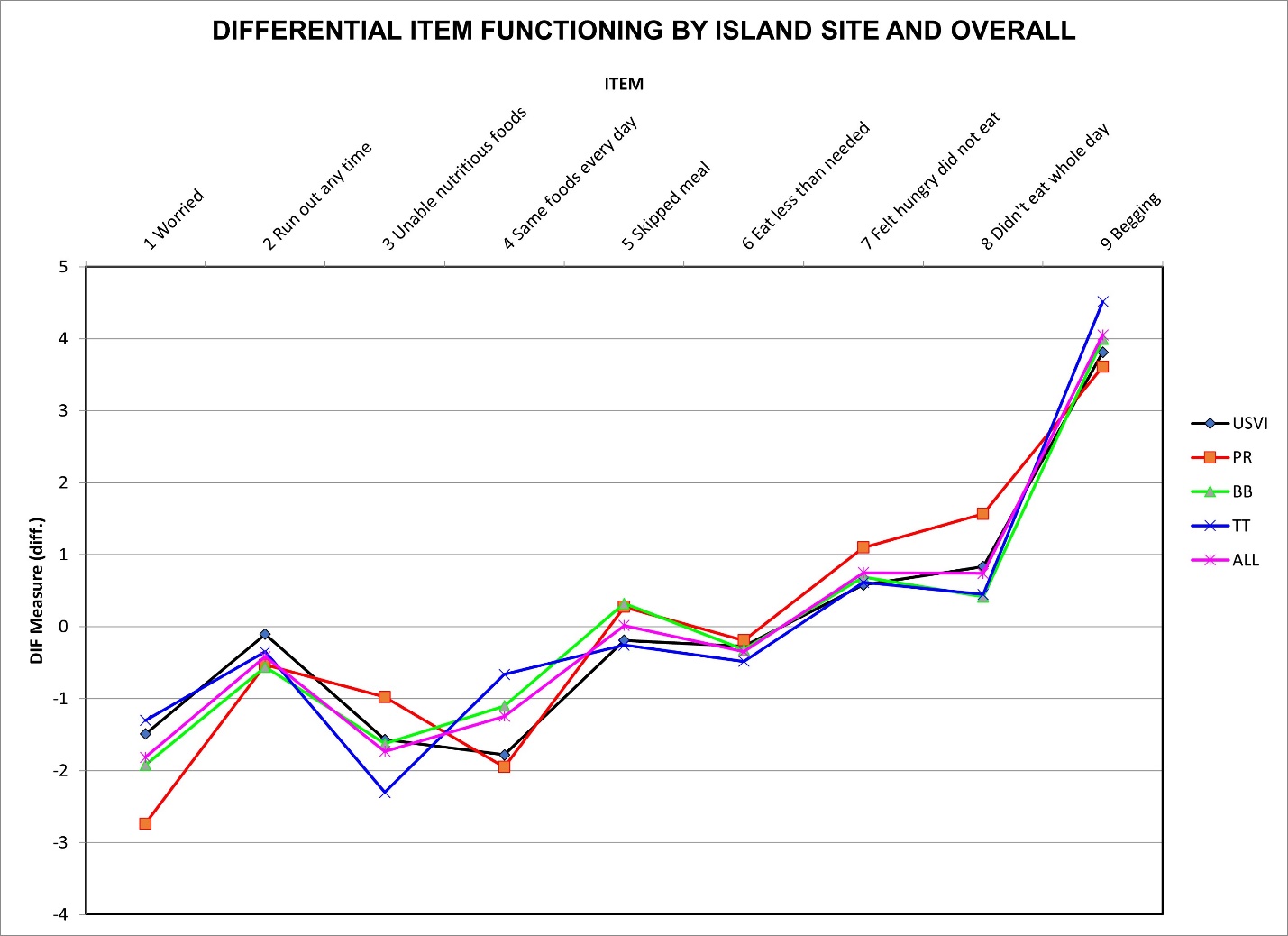
**
